# Maternal postload 1-hour glucose level during pregnancy and offspring’s overweight/obesity status in preschool age

**DOI:** 10.1136/bmjdrc-2019-000738

**Published:** 2020-02-11

**Authors:** Xiulin Shi, Peiying Huang, Liying Wang, Wei Lu, Weijuan Su, Bing Yan, Changqin Liu, Fangsen Xiao, Haiqu Song, Mingzhu Lin, Xuejun Li

**Affiliations:** 1 Xiamen Diabetes Institute, Xiamen, China; 2 Department of Endocrinology and Diabetes, Xiamen University and Fujian Medical University Affiliated First Hospital, Xiamen, China

**Keywords:** obesity, offspring, gestational diabetes mellitus, body mass index

## Abstract

**Background:**

Childhood obesity is associated with adverse outcomes such as metabolic syndrome, diabetes, and cardiovascular diseases in adulthood. Identifying risk factors related to excessive adiposity in early childhood is of great importance for obesity intervention. The results of studies for associations between maternal with gestational diabetes and offspring obesity are conflicting. Nonetheless, the association of maternal glucose across a spectrum of glucose values with childhood adiposity outcomes is less clear.

**Aim:**

To assess the association of maternal glucose across a spectrum of glucose values with childhood adiposity at age 5 years.

**Methods:**

A population-based cohort study was conducted between 2011 and 2018. Using the healthcare records data were from the Medical Birth Registry in Xiamen, China. The primary outcome was offspring obese/obesity. Primary predictors were maternal oral glucose tolerance test values during pregnancy.

**Results:**

6090 mother–child pairs were analyzed. The mean age of the children at follow-up was 5.2 years. At multiple logistic regression, after adjustment for variables, including maternal pre-pregnancy body mass index (BMI), birth weight of offspring, and insulin therapy, ORs for offspring overweight/obesity were 1.13 (95% CI 0.90 to 1.42) for maternal fasting glucose levels, 1.12 (95% CI 1.04 to 1.22) for 1-hour glucose, and 1.04 (95% CI 0.95 to 1.14) for 2-hour glucose. The adjusted association of offspring BMI Z-score with maternal 1-hour glucose level remained significant. There were no significant associations between BMI Z-score and maternal fasting glucose and 2-hour glucose level. Exploratory sex-specific analyses indicated generally consistent associations for boys and girls.

**Conclusion:**

Maternal postload 1-hour glucose across a spectrum of glucose values during pregnancy was an independent risk for offspring weight gain at age 5 years, indicating the importance of screen and management of maternal 1-hour glucose level, except for fasting glucose and 2-hour glucose level during pregnancy in order to prevent offspring weight gain in early childhood.

**Significance of this study**

**What is already known about this subject?**

- Identifying risk factors related to excessive adiposity in early childhood is of great importance for obesity intervention.
- The association of maternal gestational diabetes with offspring obesity has been investigated, findings are conflicting. Nonetheless, the association of maternal glucose across a spectrum of glucose values with childhood adiposity outcomes is unknown.

**What are the new findings?**

- After adjustment for variables, including maternal pre-pregnancy body mass index (BMI), birth weight of children, and insulin therapy, the positive association between maternal 1-hour glucose during pregnancy and measures of child adiposity is significant.
- These associations between maternal fasting glucose level and measures of child adiposity were attenuated following adjustment for maternal pre-pregnancy BMI.
- The maternal 2-hour glucose level was not associated significantly with measures of child adiposity.

**How might these results change the focus of research or clinical practice?**

- Maternal postload 1-hour glucose during pregnancy was an independent risk for offspring weight gain at age 5 years, indicating the importance of screen and management of maternal 1-hour glucose level, except for fasting glucose and 2-hour glucose level during pregnancy in order to prevent offspring weight gain in early childhood.

## Background

The prevalence of childhood obesity remains increasing in recent decades, which is alarming because it is also associated with health consequences such as metabolic syndrome, diabetes, cardiovascular diseases and even many types of cancers in adulthood.^1 2^ Once a child is obese that is more likely to remain during adulthood.^3–5^ With normal growth, children’s weight rises in proportion to height at an average age of 6 years, which is called adiposity rebound,^6–8^ and is thought to be a critical time of risk for adult obesity: obesity in this childhood period strongly predicts adult obesity.^5 9–11^ Recently, a study which included 51 000 individuals showed that ~90% of children with obesity at age 3 years had overweight or obesity by later adolescence. The most rapid increase in body mass index (BMI) in these individuals had occurred between 2 and 6 years of age. The authors concluded that the critical age for the development of sustained obesity is during early childhood.^12^ Therefore, identifying risk factors related to excessive adiposity in early childhood is of great importance for obesity intervention.

Accumulating data suggest that exposure to hyperglycemia in utero, as occurs in gestational diabetes mellitus (GDM), may expose offspring to a lifelong increased risk of obesity.^13 14^ Although maternal diagnoses of type 2 diabetes mellitus (T2D) or gestational diabetes with offspring obesity have been investigated in a number of epidemiological studies,^14–17^ findings are conflicting, a binary diagnostic classification does not capture the wide spectrum of abnormal glucose metabolism within individuals who do not display overt disease. Nonetheless, studies of the association of maternal glucose across a spectrum of glucose values with childhood adiposity outcomes remain sparse. The Hyperglycemia and Adverse Pregnancy Outcome (HAPO) cohort study provided new evidence for a continuous relationship between maternal glucose levels during pregnancy and childhood adiposity outcomes, which was independent of maternal BMI in children aged 10–14 years.^18^ The relationship between maternal glucose levels during pregnancy and offspring obesity in early childhood is currently unknown.

In this study, we aimed to assess the association of maternal glucose across a spectrum of glucose values with childhood adiposity at age 5 years

## Participants and methods

### Study design and population

This study was an evaluation of the association between maternal glucose levels during pregnancy and childhood adiposity outcomes in preschool age children. The data of both mothers and their infants were collected from the Medical Birth Registry of Xiamen (MBRX), China, between January 2011 and March 2018. The MBRX was established in 2007 and is based on a compulsory notification of all live births and stillbirths from 12 weeks’ gestation. Eligibility criteria included the following: the oral glucose tolerance test (OGTT) was conducted between 24 and 28 weeks’ gestation among mothers; gestational age at delivery ≥37 weeks and no major neonatal malformations or fetal/neonatal death; the offspring was followed up at age of 5.0 years (±6 months).

A total of 7035 mother–child pair healthcare records were available. In the present study, we included 6090 mother–child pairs (86%), after excluding the mother–child pairs missing mother’s weight or height information of pre-pregnancy (n=215), missing children’s anthropometric data from 5 years of age (n=467), and multiple births (n=56). We also excluded 81 participants reporting their medical history of diabetes (diagnosed before the index pregnancy) in this study and another 126 participants whose fasting glucose was ≥7.0 mmol/L before 12 gestational weeks because they were possible to be underdiagnosed diabetes cases before pregnancy.

All women were registered at their community health centers every time they get pregnant and then referred to a secondary hospital or a tertiary hospital for healthcare from the 32nd gestational week until delivery. All children were given the health examinations at birth (<3 days after birth), and then annual examinations until 6 years of age. Every woman and offspring were linked by individual record linkages to the Xiamen citizen health information system using the person’s unique identification number assigned to each Xiamen citizen. Every offspring was also linked with his/her biological mother’s maternal identification number.

The MBRX for pregnant women begins within the first 12 weeks of pregnancy, containing information on maternal characteristics (age at menarche, maternal age, education, BMI, obstetric history, insulin therapy, and so on); pregnancy, labor, and delivery characteristics (gestational diabetes, gestational weight gain, gestational age at delivery, hypertension in pregnancy, and so on). The MBRX for children begins with children’s birth, including information from newborns to preschool (date of birth, sex, gestational week of birth, weight, Apgar score, family history of diseases, feeding modalities, and so on). This study was carried out in accordance with the rules of the Declaration of Helsinki of 1975, revised in 2013.

### Measurements

Using the standardized protocol, all mothers’ height and weight were measured in light indoor clothing and without shoes by trained practitioners in community health centers during the pregnancy. Children’s height and weight were measured using a wall-mounted stadiometer, each child’s height was measured as length before age 2 and as standing height to the nearest 0.1 cm after age 2. Body weight was measured in kilograms using regularly calibrated electronic scales.

In China, all mothers without diagnosed diabetes were screened for GDM by a one-step approach undergoing a 75 g OGTT after fasting overnight between 24 and 28 weeks’ gestation according to the guideline from Obstetrics and Gynecology Branch of Chinese Medical Association.^19^ All mothers without diagnosed diabetes in our study were advised to undergo a 75 g OGTT after fasting overnight. Fasting, 1-hour, and 2-hour plasma glucose were measured. These continuous variables were also divided into discrete categorical variables with four categories for each measure. Categories were defined as follows: fasting glucose ≤4.1, 4.2–4.4, 4.5–4.6, ≥4.7 mmol/L; 1-hour glucose ≤6.9, 7.0–8.0, 8.1–9.1, ≥9.2 mmol/L; 2-hour glucose ≤5.9, 6.0–6.7, 6.8–7.6, ≥7.7 mmol/L.

### Children’s Z-scores for BMI and childhood overweight/obesity

BMI Z-score for age was used to describe the change in the offspring’s BMI. We calculated sex-adjusted and age-adjusted Z-score of childhood BMI using Chinese reference growth charts.^20^ Childhood overweight was defined as a BMI at or above the 85th percentile and below the 95th percentile, and obesity was defined as a BMI at or above the 95th percentile.

### Statistical methods

Data were reported using frequencies and counts for categorical variables and means and SDs for continuous variables. Multiple logistic regression was used for dichotomous outcomes and results are reported as ORs with 95% CI. Multiple linear regression was used for continuous outcomes and results are reported as regression coefficients (β estimates) with 95% CI. Four multivariable-adjusted models were included in these analyses: model 1 adjusted for maternal age, education, offspring sex, and infant feeding; model 2 adjusted for the variables in model 1 plus maternal pre-pregnancy BMI; model 3 adjusted for the variables in model 2 plus offspring birth weight; model 4 adjusted for the variables in model 3 plus insulin therapy. Exploratory analyses were also conducted to evaluate differences in associations according to sex. Significance tests were two tailed and a p value <0.05 was considered statistically significant. The data analysis for this article was generated using SAS V.9.4 for the Windows x64-based system.

## Results

A total of 7035 mother–child pair healthcare records were available and 6090 mother–child pairs (86%) were analyzed. By comparing the main characteristics, we found no difference between the included and excluded population. The specific characteristics of the excluded and included groups for mothers were as follows: maternal age before pregnancy: 27.2 (3.4) vs 27.2 (3.3), p=0.514; pre-pregnancy BMI: 20.9 (2.7) vs 20.9 (2.7), p=0.236; gestational weight gain: 12.7 (4.4) vs 12.7 (4.4), p=0.693; fasting plasma glucose level: 4.4 (0.4) vs 4.4 (0.4), p=0.214; 1-hour plasma glucose level: 8.0 (1.6) vs 8.0 (1.6), p=0.212; 2-hour plasma glucose level: 6.8 (1.3) vs 6.8 (1.3), p=0.718. The specific characteristics of the excluded and included groups for offspring were as follows: birth weight: 3.2 (0.7) vs 3.2 (0.7), p=0.889. The general maternal and child characteristics are presented (table 1). The mean maternal age before pregnancy was 27.2 years. The mean pre-pregnancy BMI was 20.9 kg/m^2^. The mean fasting, 1-hour, and 2-hour glucose level during the OGTT was 4.4 mmol/L, 8.0 mmol/L and 6.8 mmol/L, respectively. The mean birth weight of children was 3.2 kg. The mean age of the children at follow-up was 5.2 years.

**Table 1:** Characteristics of mothers and their children

Variable	n=6090
Maternal characteristics	
Maternal age before pregnancy, mean (SD), years	27.2 (3.4)
Gestational age at delivery, mean (SD), weeks	38.9 (1.7)
Pre-pregnancy BMI, mean (SD), kg/m^2^	20.9 (2.7)
Gestational weight gain, mean (SD), kg	12.7 (4.4)
Mean systolic pressure, mean (SD), mm Hg	105.5 (10.9)
Mean diastolic pressure, mean (SD), mm Hg	66.0 (7.9)
Fasting plasma glucose level, mean (SD), mmol/L	4.4 (0.4)
1-hour plasma glucose level, mean (SD), mmol/L	8.0 (1.6)
2-hour plasma glucose level, mean (SD), mmol/L	6.8 (1.3)
Child characteristics	
Boy, n (%)	3287 (53.8)
Birth weight (kg)	3.2 (0.7)
Mode of infant feeding within the first 6 months, n (%)	
Exclusive formula feeding	1090 (20.7)
Exclusive breast feeding	3812 (72.4)
Mixed breast and formula	365 (6.9)
Age at follow-up	5.2 (0.4)
BMI Z-scores at follow-up, mean (SD)	−0.05 (0.9)

BMI, body mass index.

Linear associations of continuous offspring BMI Z-score with maternal fasting, 1-hour, and 2-hour glucose level during pregnancy were initially analyzed using multiple linear regression (table 2). In model 1, positive associations for continuous offspring BMI Z-score were observed for 1 mmol/L differences in maternal fasting glucose measure. The association was null after adjustment for maternal pre-pregnancy BMI. In models 1–4, the association between offspring BMI Z-score with maternal 1-hour glucose level was still significant after adjustment for maternal pre-pregnancy BMI, offspring birth weight, and insulin therapy. The offspring BMI Z-score was not associated significantly with maternal 2-hour glucose level in models 1–4.

**Table 2:** Associations of continuous measures of maternal glucose during pregnancy with adiposity outcomes among children at follow-up

Outcome	BMI Z-score		Overweight/obese	
	β (95% CI)	P value	OR (95% CI)	P value
Fasting plasma glucose				
Model 1	0.15 (0.08 to 0.22)	<0.0001***	1.41 (1.14 to 1.75)	0.0019**
Model 2	0.06 (−0.02 to 0.13)	0.14	1.12 (0.90 to 1.40)	0.30
Model 3	0.05 (−0.02 to 0.12)	0.19	1.12 (0.89 to 1.40)	0.32
Model 4	0.05 (−0.02 to 0.12)	0.19	1.13 (0.90 to 1.42)	0.29
1-hour plasma glucose				
Model 1	0.04 (0.02 to 0.06)	<0.0001***	1.14 (1.08 to 1.21)	<0.0001***
Model 2	0.02 (0.003 to 0.04)	0.02*	1.10 (1.10 to 1.17)	0.002***
Model 3	0.02 (0.004 to 0.04)	0.02*	1.10 (1.03 to 1.17)	0.002***
Model 4	0.02 (0.00 to 0.04)	0.01*	1.12 (1.04 to 1.22)	0.003***
2-hour plasma glucose				
Model 1	0.041 (−0.02 to 0.03)	0.72	1.06 (0.99 to 1.13)	0.11
Model 2	−0.008 (−0.03 to 0.02)	0.49	1.03 (0.60 to 1.10)	0.43
Model 3	−0.008 (−0.03 to 0.01)	0.47	1.02 (0.96 to 1.10)	0.50
Model 4	−0.01 (−0.03 to 0.01)	0.43	1.04 (0.95 to 1.14)	0.41

Model 1: adjusted for maternal age, education, offspring sex, and infant feeding.

Model 2: adjusted for covariates in model 1+maternal pre-pregnancy body mass index.

Model 3: adjusted for covariates in model 2+birth weight.

Model 4: adjusted for covariates in model 3+insulin therapy.

*P< 0.05; **p<0.01; ***p<0.001.

BMI, body mass index.

The association of maternal glucose across the continuum with dichotomous offspring overweight/obesity was also evaluated using multiple logistic regression. ORs for offspring overweight/obesity were estimated for 1 mmol/L differences in maternal fasting, 1-hour and 2-hour glucose level during pregnancy (table 2). In model 1, positive associations for offspring overweight/obesity were observed for 1 mmol/L differences in maternal fasting glucose measure, and OR was 1.41 (95% CI 1.14 to 1.75). ORs were attenuated after adjusting for maternal pre-pregnancy BMI. In models 1–4, the association between offspring overweight/obesity and maternal 1-hour glucose level was significant after adjustment for maternal pre-pregnancy BMI, offspring birth weight, and insulin therapy, and OR was 1.14 (95% CI 1.08 to 1.21) in model 1; 1.10 (95% CI 1.10 to 1.17) for adjustment for maternal pre-pregnancy BMI, 1.10 (95% CI 1.03 to 1.17) for adjustment for offspring birth, and 1.12 (95% CI 1.04 to 1.22) for adjustment for insulin therapy, respectively. The offspring overweight/obesity was not associated significantly with maternal 2-hour glucose level in models 1–4.

**Figure 1:**
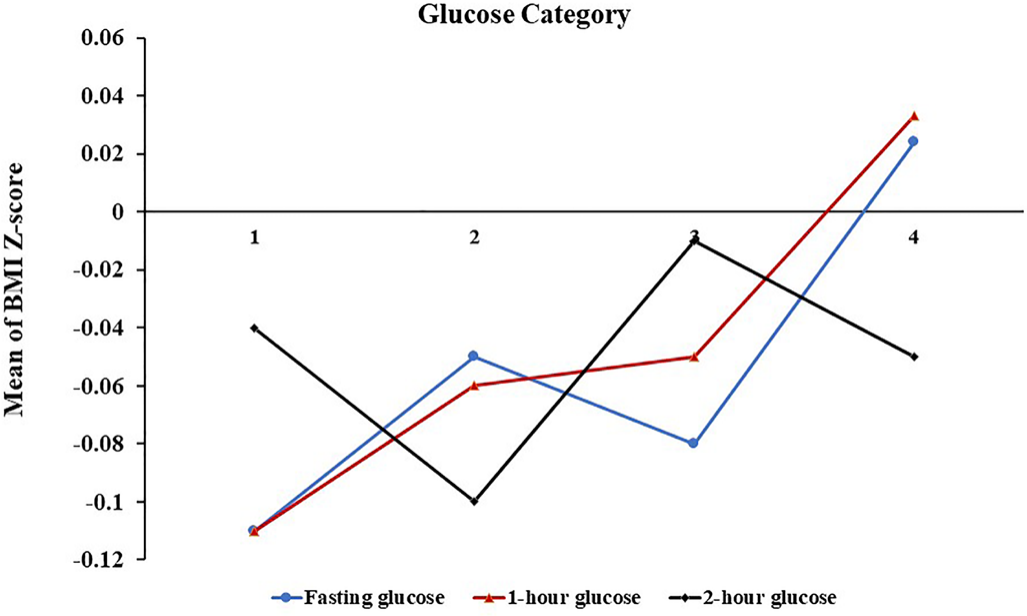
Mean of offspring BMI Z-score across categories of maternal fasting glucose (blue line), 1-hour glucose (red line), and 2-hour glucose (black line). Glucose categories were on the x-axis. Glucose categories are defined as follows: fasting glucose level: category 1, ≤4.1 mmol/L; category 2, 4.2–4.4 mmol/L; category 3, 4.5–4.6 mmol/L; category 4, ≥4.7 mmol/L; 1-hour glucose level: category 1, ≤6.9 mmol/L; category 2, 7.0–8.0 mmol/L; category 3, 8.1–9.1 mmol/L; category 4, ≥9.2 mmol/L; and 2-hour glucose level: category 1, ≤5.9 mmol/L; category 2, 6.0–6.7 mmol/L; category 3, 6.8–7.6 mmol/L; category 4, ≥7.7 mmol/L. BMI, body mass index.

**Figure 2:**
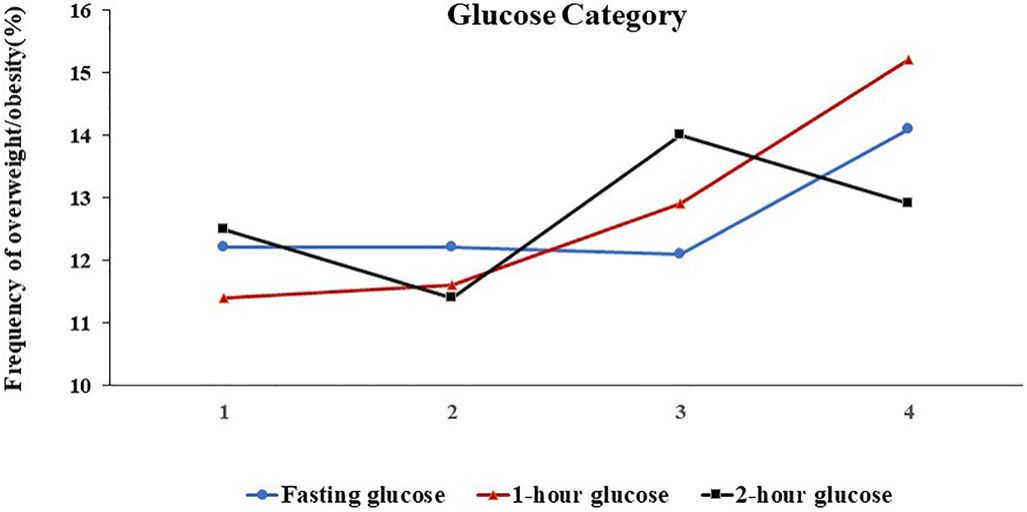
Frequency of offspring overweight/obesity across categories of maternal fasting glucose (blue line), 1-hour glucose (red line), and 2-hour glucose (black line). Glucose categories were on the x-axis. Glucose categories are defined as follows: fasting glucose level: category 1, ≤4.1 mmol/L; category 2, 4.2–4.4 mmol/L; category 3, 4.5–4.6 mmol/L; category 4, ≥4.7 mmol/L; 1-hour glucose level: category 1, ≤6.9 mmol/L; category 2, 7.0–8.0 mmol/L; category 3, 8.1–9.1 mmol/L; category 4, ≥9.2 mmol/L; and 2-hour glucose level: category 1, ≤5.9 mmol/L; category 2, 6.0–6.7 mmol/L; category 3, 6.8–7.6 mmol/L; category 4, ≥7.7 mmol/L.

The offspring BMI Z-score and the frequency of overweight/obesity across the four glucose categories were shown in figures 1 and 2. After multivariate adjustment, only with increasing maternal 1-hour glucose levels, these outcomes significantly increased. For the 1-hour glucose level, the outcome in the lowest and highest categories, respectively, was −0.11 and 0.03 for the mean of BMI Z-score, 11.4% and 15.2% for the frequency of overweight/obesity (p_trend_<0.01).

Exploratory analyses were performed to evaluate sex-specific associations. Statistical interaction terms for offspring BMI Z-score and overweight/obesity confirmed generally consistent associations for boys and girls (p>0.05) (table 3).

**Table 3:** Sex-specific fully adjusted associations of continuous measures of maternal glucose during pregnancy with adiposity outcomes among children at follow-up

Outcome	Boys		Girls		Interaction term P value‡
	(95% CI)	P value*	(95% CI)	P value†	
BMI Z-score β (95% CI)					
Fasting plasma glucose	0.06 (−0.05 to 0.17)	0.31	0.02 (−0.07 to 0.11)	0.63	0.39
1-hour plasma glucose	0.02 (−0.005 to 0.05)	0.11	0.02 (0.004 to 0.04)	0.02	0.57
2-hour plasma glucose	−0.03 (−0.06 to 0.002)	0.07	−0.014 (−0.015 to 0.04)	0.34	0.10
Overweight/obese OR (95% CI)					
Fasting plasma glucose	1.14 (0.86 to 1.51)	0.36	1.10 (0.75 to 1.60)	0.24	0.55
1-hour plasma glucose	1.07 (0.99 to 1.16)	0.07	1.17 (1.03 to 1.17)	0.006**	0.73
2-hour plasma glucose	0.96 (0.88 to 1.05)	0.35	1.13 (1.00 to 1.27)	0.045*	0.14

Models are adjusted for maternal age, education, offspring sex, infant feeding, maternal pre-pregnancy body mass index, birth weight, and insulin therapy.

*P value for boys only; *p<0.05; **p<0.01.

†P value for girls only; *p<0.05; **p<0.01.

‡P value for sex * maternal predictor interaction term including boys and girls; *p<0.05.

BMI, body mass index.

## Discussion

In this retrospective cohort study among 6090 mother–child pairs with mean 5 years of follow-up, we found that the positive association between maternal 1-hour glucose during pregnancy and measures of child adiposity is present across the spectrum of maternal glucose levels during pregnancy after adjustment for variables, including maternal pre-pregnancy BMI, birth weight of children, and insulin therapy. These associations between maternal fasting glucose level and measures of child adiposity were attenuated following adjustment for maternal pre-pregnancy BMI. The maternal 2-hour glucose level was not associated significantly with measures of child adiposity. Exploratory sex-specific analyses indicated generally consistent associations for boys and girls. These results indicate the importance of measurement and control of 1-hour glucose level for mothers during pregnancy in order to prevent offspring weight gain in early childhood.

Recent smaller studies of children reported positive associations between maternal glucose levels during pregnancy and measures of adiposity.^21 22^ The HAPO study also demonstrated continuous associations between maternal glucose levels during pregnancy and newborn and offspring aged 10–14 years’ adiposity outcomes.^18 23^ Our study found that the positive association between maternal 1-hour glucose level of OGTT during pregnancy and measures of child adiposity is present across the spectrum of maternal glucose levels during pregnancy after adjustment for variables, including maternal pre-pregnancy BMI, birth weight of children and insulin therapy. Our study demonstrated that maternal 1-hour glucose level of OGTT during pregnancy was an independent risk for offspring weight gain. The previous metabolomic studies in the HAPO cohort demonstrated associations of maternal 1-hour glucose levels with maternal serum levels of triacylglycerol, non-esterified fatty acids, β-hydroxybutyrate, and several amino acids,^24^ which are important contributors to excess fetal growth and fat accretion.^25^ However, in clinical practice in China, doctors and mothers with GDM or T2D pay more attention to the control of fasting blood glucose and 2-hour postprandial blood glucose, and often ignore monitoring and control of 1-hour glucose level after a meal. In our study, after adjustment for insulin therapy, the association between maternal 1-hour glucose level of OGTT and offspring overweight/obesity was still significant, indicating ignore of the control of 1-hour glucose level in maternal may reduce the effect of insulin therapy on offspring outcome. Jovanovic-Peterson *et al* 26 have suggested monitoring of non-fasting glucose levels rather than the fasting levels, which are more commonly monitored in clinical practice, is necessary to prevent macrosomia. The results of previous studies suggested that treatment of gestational diabetes reduces serious perinatal morbidity and the rate of macrosomia.^27 28^

In our study, statistical interaction terms confirmed generally consistent associations for boys and girls. Differences in adiposity between girls and boys have been previously reported. At birth, percent body fat is higher in girls than in boys,^29^ and at the age of 10 years, the OR estimate for obesity with higher levels of maternal fasting glucose was higher in girls.^18^ Among HAPO participants from Hong Kong, maternal glucose levels during the HAPO OGTT were associated with being overweight and adiposity in girls but not boys.^21^ However, one study has reported that GDM was associated with higher fat mass in boys but not girls at age ~8 years.^30^

This study has several strengths. First, measurement of offspring height, weight, and confounders such as maternal age and weight gain was conducted by a trained nurse. Second, insulin therapy was adjusted in this study of childhood outcomes. Third, data are based on China, identification and understanding of early life risk factors are particularly urgent because of the increasing burden of childhood obesity and diabetes.

There are some limitations to our study. First, there are not some childhood lifestyle factors, such as physical activity and nutrition in this study, since we used healthcare records as a main source of data. Second, most of the participants were Chinese, we should be cautious when generalizing the findings.

## Conclusions

Our study shows new evidence for a continuous relationship between maternal 1-hour glucose levels during pregnancy and childhood adiposity outcomes that is independent of maternal BMI, offspring birth weight and insulin therapy, indicating the importance of screen and management of maternal 1-hour glucose level, except for fasting glucose and 2-hour glucose level during pregnancy in order to prevent offspring weight gain in early childhood.
